# Correction to: Changes in self-harm- and violence-related urgent psychiatric consultation in the emergency department during the different stages of the COVID-19 pandemic

**DOI:** 10.1186/s12888-023-04652-9

**Published:** 2023-03-09

**Authors:** Chun Hao Liu, Po-Cheng Chen, Jian Hong Chen, Chung Cheng Yeh

**Affiliations:** 1 Department of Child Psychiatry, Chang Gung Memorial Hospital at Linkou, Taoyuan, Taiwan; 2 College of Medicine, Chang Gung University, Taoyuan, Taiwan; 3 Department of Rehabilitation Medicine, Kaohsiung Chang Gung Memorial Hospital, Kaohsiung, Taiwan; 4 Department of Psychiatry, Chang Gung Memorial Hospital at Keelung, Keelung, Taiwan; 5 National Taiwan Sport University, Taoyuan, Taiwan; 6 Department of Emergency Medicine, Chang Gung Memorial Hospital at Keelung, Keelung, Taiwan

Following publication of the original article [1], the authors identified an error in the author name of Po-Cheng Chen.

The incorrect author name is: Po-Chen Chen.

The correct author name is: Po-Cheng Chen.

The author group has been updated above and the original article [1] has been corrected.
